# 4-Bromo-2-[(*E*)-(4-chloro­phen­yl)imino­meth­yl]phenol

**DOI:** 10.1107/S1600536811004417

**Published:** 2011-02-12

**Authors:** Amir Adabi Ardakani, Reza Kia, Hadi Kargar, Muhammad Nawaz Tahir

**Affiliations:** a Islamic Azad University, Ardakan Branch, Iran; b X-ray Crystallography Laboratory, Plasma Physics Research Center, Science and Research Branch, Islamic Azad University, Tehran, Iran; c Chemistry Department, Payame Noor University, Tehran 19395-4697, I. R. of Iran; d Department of Physics, University of Sargodha, Punjab, Pakistan

## Abstract

In the title compound, C_13_H_9_BrClNO, the dihedral angle between the substituted benzene rings is 43.90 (11)°. Strong intra­molecular O—H⋯N hydrogen bonds generate *S*(6) ring motifs. The crystal structure features short intemolecular Br⋯Br [3.554 (2) Å] and Cl⋯Cl [3.412 (2) Å] contacts. The crystal packing is further stabilized by inter­molecular C—H⋯O and C—H⋯π inter­actions.

## Related literature

For standard bond lengths, see: Allen *et al.* (1987 ▶). For hydrogen-bond motifs, see: Bernstein *et al.* (1995 ▶). For van der Waals radii, see: Bondi (1964 ▶).
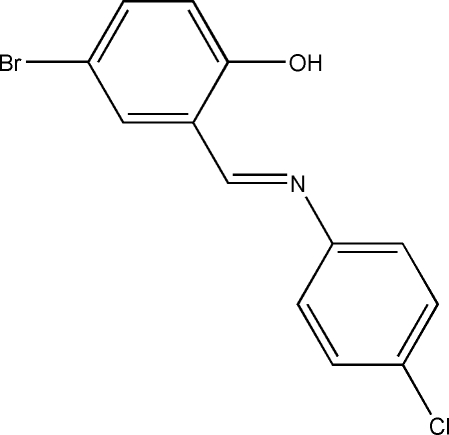


## Experimental

### 

#### Crystal data

C_13_H_9_BrClNO
*M* *_r_* = 310.57
Monoclinic, 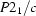

*a* = 27.652 (11) Å
*b* = 7.011 (3) Å
*c* = 6.219 (3) Å
β = 96.38 (2)°
*V* = 1198.2 (8) Å^3^
*Z* = 4
Mo *K*α radiation
μ = 3.63 mm^−1^
*T* = 296 K
0.35 × 0.25 × 0.22 mm

#### Data collection

Bruker SMART APEXII CCD area-detector diffractometer
Absorption correction: multi-scan (*SADABS*; Bruker, 2005 ▶) *T*_min_ = 0.363, *T*_max_ = 0.502
5719 measured reflections
2170 independent reflections
1718 reflections with *I* > 2σ(*I*)
*R*_int_ = 0.026

#### Refinement

*R*[*F* ^2^ > 2σ(*F* ^2^)] = 0.031
*wR*(*F* ^2^) = 0.078
*S* = 1.02
2170 reflections
155 parameters
H-atom parameters constrained
Δρ_max_ = 0.39 e Å^−3^
Δρ_min_ = −0.35 e Å^−3^

### 

Data collection: *APEX2* (Bruker, 2005 ▶); cell refinement: *SAINT* (Bruker, 2005 ▶); data reduction: *SAINT*; program(s) used to solve structure: *SHELXTL* (Sheldrick, 2008 ▶); program(s) used to refine structure: *SHELXTL*; molecular graphics: *SHELXTL*; software used to prepare material for publication: *SHELXTL* and *PLATON* (Spek, 2009 ▶).

## Supplementary Material

Crystal structure: contains datablocks global, I. DOI: 10.1107/S1600536811004417/jh2265sup1.cif

Structure factors: contains datablocks I. DOI: 10.1107/S1600536811004417/jh2265Isup2.hkl

Additional supplementary materials:  crystallographic information; 3D view; checkCIF report

## Figures and Tables

**Table 1:** Hydrogen-bond geometry (Å, °) *Cg*1 is the centroid of the C1–C6 benzene ring.

*D*—H⋯*A*	*D*—H	H⋯*A*	*D*⋯*A*	*D*—H⋯*A*
O1—H1⋯N1	0.82	1.87	2.594 (3)	147
C9—H9⋯O1^i^	0.93	2.60	3.459 (4)	154
C10—H10⋯*Cg*1^ii^	0.93	2.77	3.474 (3)	134
C13—H13⋯*Cg*1^iii^	0.03	2.80	3.501 (3)	133

Symmetry codes: (i) 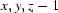; (ii) 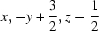; (iii) 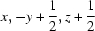.

**Fig. 1.**
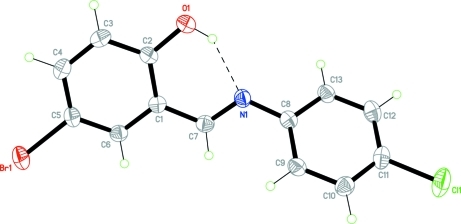
The asymmetric unit of the title compound, showing 40% probability displacement ellipsoids and the atomic numbering. Intramolecular hydrogen bonds are drawn as dashed lines.

**Fig. 2.**
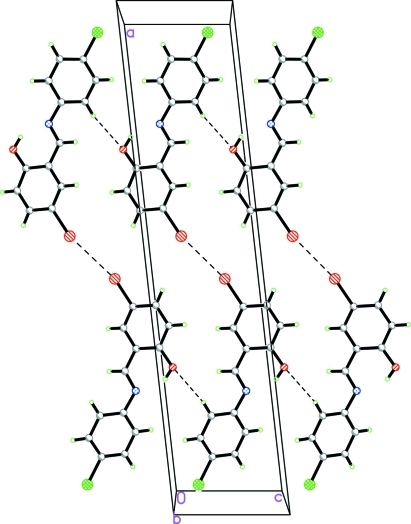
The packing diagram of the title compound, viewed down the b-axis forming sheets through the intermolecular Br···Br and C—H···O interactions. The intermolecular interactions are shown as dashed lines.

## supplementary crystallographic information

### Comment

Schiff base ligands are one of the most prevalent systems in coordination chemistry. As part of a general study of Schiff bases, we have determined the crystal structure of the title compound.

The asymmetric unit of the title compound, Fig. 1, comprises a potentially bidentate Schiff base ligand. The bond lengths (Allen *et al.,* 1987) and angles are within the normal ranges. The dihedral angle between the substituted benzene rings is 43.90 (11)Å. Strong intramolecular O—H···N hydrogen bonds generate *S(6)* ring motifs (Bernstein *et al.*, 1995). The remarkable features of the crystal structure is the intemolecular Br···Br [3.554 (2)Å] and Cl···Cl [3.412 (2)Å] contacts which are shorter than the sum of the van der Waals radii of these atoms (Bondi 1964). The crystal packing is further stabilized by the intermolecular C—H···O hydrogen bond (Table 1) and C—H···π interaction [C10—H10···*Cg*1^ii^ =  3.474 (3)Å, (ii) X, 3/2 - Y, -1/2 + Z; C13—H13···*Cg*1^iii^ = 3.501 (3)Å, (iii) X, 1/2 - Y, 1/2 + Z, *Cg*1 is the centroid of the C1–C6 benzene ring].

### Experimental

The title compound was synthesized by adding 5-bromo-salicylaldehyde (2 mmol) to a solution of *p*-chloroaniline (2 mmol) in ethanol (20 ml). The mixture was refluxed with stirring for half an hour. The resulting light-yellow solution was filtered. Light-yellow single crystals suitable for *X*-ray diffraction were recrystallized from ethanol by slow evaporation of the solvents at room temperature over several days.

### Refinement

H atoms of the hydroxy groups were located by a rotating model and constrained to refine with the parent atoms with U_iso_(H) = 1.5 U_eq_(O), see Table 1. The remaining H atoms were positioned geometrically with C—H = 0.93 Å and included in a riding model approximation with U_iso_ (H) = 1.2 U_eq_ (C).

### Crystal data

**Table:**

C_13_H_9_BrClNO	*F*(000) = 616
*M**_r_* = 310.57	*D*_x_ = 1.722 Mg m^−^^3^
Monoclinic, *P*2_1_/*c*	Mo *K*α radiation, λ = 0.71073 Å
Hall symbol: -P 2ybc	Cell parameters from 2520 reflections
*a* = 27.652 (11) Å	θ = 2.5–27.5°
*b* = 7.011 (3) Å	µ = 3.63 mm^−^^1^
*c* = 6.219 (3) Å	*T* = 296 K
β = 96.38 (2)°	Prism, light-yellow
*V* = 1198.2 (8) Å^3^	0.35 × 0.25 × 0.22 mm
*Z* = 4	

### Data collection

**Table:**

Bruker SMART APEXII CCD area-detector diffractometer	2170 independent reflections
Radiation source: fine-focus sealed tube	1718 reflections with *I* > 2σ(*I*)
graphite	*R*_int_ = 0.026
φ and ω scans	θ_max_ = 25.3°, θ_min_ = 1.5°
Absorption correction: multi-scan (*SADABS*; Bruker, 2005)	*h* = −28→33
*T*_min_ = 0.363, *T*_max_ = 0.502	*k* = −8→5
5719 measured reflections	*l* = −7→6

### Refinement

**Table:**

Refinement on *F*^2^	Primary atom site location: structure-invariant direct methods
Least-squares matrix: full	Secondary atom site location: difference Fourier map
*R*[*F*^2^ > 2σ(*F*^2^)] = 0.031	Hydrogen site location: inferred from neighbouring sites
*wR*(*F*^2^) = 0.078	H-atom parameters constrained
*S* = 1.02	*w* = 1/[σ^2^(*F*_o_^2^) + (0.0399*P*)^2^ + 0.3051*P*] where *P* = (*F*_o_^2^ + 2*F*_c_^2^)/3
2170 reflections	(Δ/σ)_max_ = 0.001
155 parameters	Δρ_max_ = 0.39 e Å^−^^3^
0 restraints	Δρ_min_ = −0.35 e Å^−^^3^

### Special details

**Table:**

Geometry. All esds (except the esd in the dihedral angle between two l.s. planes) are estimated using the full covariance matrix. The cell esds are taken into account individually in the estimation of esds in distances, angles and torsion angles; correlations between esds in cell parameters are only used when they are defined by crystal symmetry. An approximate (isotropic) treatment of cell esds is used for estimating esds involving l.s. planes.
Refinement. Refinement of F^2^ against ALL reflections. The weighted R-factor wR and goodness of fit S are based on F^2^, conventional R-factors R are based on F, with F set to zero for negative F^2^. The threshold expression of F^2^ > 2sigma(F^2^) is used only for calculating R-factors(gt) etc. and is not relevant to the choice of reflections for refinement. R-factors based on F^2^ are statistically about twice as large as those based on F, and R- factors based on ALL data will be even larger.

### Fractional atomic coordinates and isotropic or equivalent isotropic displacement parameters (Å^2^)

**Table:**

	*x*	*y*	*z*	*U*_iso_*/*U*_eq_	
Br1	0.456428 (11)	0.43271 (5)	0.67549 (5)	0.05153 (14)	
Cl1	0.03796 (3)	0.54095 (14)	0.23081 (16)	0.0684 (3)	
O1	0.27742 (7)	0.5674 (3)	1.1170 (3)	0.0444 (5)	
H1	0.2529	0.5423	1.0354	0.067*	
N1	0.22712 (8)	0.4746 (3)	0.7551 (3)	0.0313 (5)	
C1	0.31315 (10)	0.4668 (3)	0.7991 (4)	0.0289 (6)	
C2	0.31691 (10)	0.5350 (3)	1.0152 (4)	0.0309 (6)	
C3	0.36211 (11)	0.5707 (3)	1.1235 (4)	0.0361 (6)	
H3	0.3647	0.6166	1.2646	0.043*	
C4	0.40322 (11)	0.5392 (3)	1.0256 (4)	0.0363 (6)	
H4	0.4336	0.5642	1.1000	0.044*	
C5	0.39973 (10)	0.4695 (3)	0.8134 (4)	0.0331 (6)	
C6	0.35529 (10)	0.4331 (3)	0.7031 (4)	0.0304 (6)	
H6	0.3532	0.3855	0.5628	0.036*	
C7	0.26674 (10)	0.4453 (3)	0.6737 (4)	0.0311 (6)	
H7	0.2655	0.4091	0.5294	0.037*	
C8	0.18255 (10)	0.4827 (3)	0.6229 (4)	0.0298 (6)	
C9	0.17902 (10)	0.5621 (3)	0.4151 (4)	0.0332 (6)	
H9	0.2069	0.6033	0.3578	0.040*	
C10	0.13425 (11)	0.5790 (3)	0.2961 (4)	0.0365 (6)	
H10	0.1315	0.6315	0.1579	0.044*	
C11	0.09378 (11)	0.5175 (4)	0.3836 (5)	0.0390 (7)	
C12	0.09647 (11)	0.4396 (3)	0.5890 (5)	0.0405 (7)	
H12	0.0685	0.3987	0.6455	0.049*	
C13	0.14112 (10)	0.4237 (3)	0.7077 (4)	0.0334 (6)	
H13	0.1434	0.3727	0.8465	0.040*	

### Atomic displacement parameters (Å^2^)

**Table:**

	*U*^11^	*U*^22^	*U*^33^	*U*^12^	*U*^13^	*U*^23^
Br1	0.0284 (2)	0.0679 (2)	0.0599 (2)	−0.00058 (15)	0.01204 (13)	−0.00234 (15)
Cl1	0.0355 (5)	0.0899 (7)	0.0749 (6)	0.0049 (5)	−0.0157 (4)	0.0006 (5)
O1	0.0349 (12)	0.0649 (13)	0.0343 (10)	0.0008 (10)	0.0085 (8)	−0.0095 (9)
N1	0.0270 (13)	0.0334 (11)	0.0336 (11)	−0.0016 (10)	0.0041 (9)	0.0012 (9)
C1	0.0319 (16)	0.0250 (12)	0.0299 (13)	0.0014 (11)	0.0035 (10)	0.0002 (10)
C2	0.0323 (16)	0.0308 (13)	0.0305 (13)	0.0023 (12)	0.0080 (11)	0.0014 (11)
C3	0.0433 (18)	0.0353 (14)	0.0284 (13)	−0.0017 (13)	−0.0015 (11)	−0.0030 (11)
C4	0.0323 (17)	0.0362 (14)	0.0386 (14)	−0.0030 (12)	−0.0045 (12)	0.0012 (11)
C5	0.0273 (16)	0.0307 (13)	0.0417 (14)	0.0010 (11)	0.0055 (11)	0.0044 (11)
C6	0.0331 (16)	0.0297 (13)	0.0285 (12)	0.0022 (12)	0.0034 (10)	−0.0002 (10)
C7	0.0343 (16)	0.0293 (13)	0.0300 (12)	−0.0008 (11)	0.0043 (11)	−0.0025 (10)
C8	0.0314 (16)	0.0249 (12)	0.0336 (13)	0.0012 (11)	0.0057 (11)	−0.0015 (10)
C9	0.0321 (16)	0.0346 (14)	0.0338 (13)	−0.0017 (12)	0.0083 (11)	0.0022 (11)
C10	0.0381 (18)	0.0351 (14)	0.0358 (14)	0.0041 (12)	0.0014 (12)	0.0011 (11)
C11	0.0291 (17)	0.0362 (14)	0.0502 (16)	0.0038 (13)	−0.0029 (13)	−0.0057 (13)
C12	0.0298 (17)	0.0396 (15)	0.0531 (17)	−0.0042 (13)	0.0095 (13)	−0.0017 (13)
C13	0.0308 (16)	0.0348 (14)	0.0358 (13)	−0.0013 (12)	0.0087 (11)	0.0036 (11)

### Geometric parameters (Å, °)

**Table:**

Br1—C5	1.886 (3)	C5—C6	1.363 (4)
Cl1—C11	1.728 (3)	C6—H6	0.9300
O1—C2	1.341 (3)	C7—H7	0.9300
O1—H1	0.8200	C8—C13	1.377 (4)
N1—C7	1.273 (3)	C8—C9	1.400 (3)
N1—C8	1.405 (3)	C9—C10	1.375 (4)
C1—C6	1.387 (4)	C9—H9	0.9300
C1—C2	1.420 (3)	C10—C11	1.367 (4)
C1—C7	1.434 (4)	C10—H10	0.9300
C2—C3	1.375 (4)	C11—C12	1.384 (4)
C3—C4	1.366 (4)	C12—C13	1.371 (4)
C3—H3	0.9300	C12—H12	0.9300
C4—C5	1.401 (4)	C13—H13	0.9300
C4—H4	0.9300		
			
C2—O1—H1	109.5	N1—C7—H7	119.2
C7—N1—C8	120.8 (2)	C1—C7—H7	119.2
C6—C1—C2	119.2 (2)	C13—C8—C9	119.8 (3)
C6—C1—C7	119.5 (2)	C13—C8—N1	118.5 (2)
C2—C1—C7	121.1 (2)	C9—C8—N1	121.5 (2)
O1—C2—C3	118.8 (2)	C10—C9—C8	119.8 (3)
O1—C2—C1	121.7 (2)	C10—C9—H9	120.1
C3—C2—C1	119.5 (3)	C8—C9—H9	120.1
C4—C3—C2	120.6 (2)	C11—C10—C9	119.1 (3)
C4—C3—H3	119.7	C11—C10—H10	120.4
C2—C3—H3	119.7	C9—C10—H10	120.4
C3—C4—C5	120.2 (3)	C10—C11—C12	122.0 (3)
C3—C4—H4	119.9	C10—C11—Cl1	118.1 (2)
C5—C4—H4	119.9	C12—C11—Cl1	119.9 (2)
C6—C5—C4	120.2 (3)	C13—C12—C11	118.8 (3)
C6—C5—Br1	119.6 (2)	C13—C12—H12	120.6
C4—C5—Br1	120.2 (2)	C11—C12—H12	120.6
C5—C6—C1	120.3 (2)	C12—C13—C8	120.5 (2)
C5—C6—H6	119.8	C12—C13—H13	119.8
C1—C6—H6	119.8	C8—C13—H13	119.8
N1—C7—C1	121.7 (2)		
			
C6—C1—C2—O1	−179.0 (2)	C6—C1—C7—N1	180.0 (2)
C7—C1—C2—O1	5.5 (4)	C2—C1—C7—N1	−4.6 (4)
C6—C1—C2—C3	1.5 (3)	C7—N1—C8—C13	149.2 (2)
C7—C1—C2—C3	−173.9 (2)	C7—N1—C8—C9	−35.6 (3)
O1—C2—C3—C4	179.9 (2)	C13—C8—C9—C10	−0.6 (4)
C1—C2—C3—C4	−0.7 (4)	N1—C8—C9—C10	−175.7 (2)
C2—C3—C4—C5	−0.2 (4)	C8—C9—C10—C11	0.0 (4)
C3—C4—C5—C6	0.2 (4)	C9—C10—C11—C12	0.3 (4)
C3—C4—C5—Br1	178.26 (19)	C9—C10—C11—Cl1	179.82 (19)
C4—C5—C6—C1	0.7 (4)	C10—C11—C12—C13	0.0 (4)
Br1—C5—C6—C1	−177.37 (17)	Cl1—C11—C12—C13	−179.53 (19)
C2—C1—C6—C5	−1.6 (4)	C11—C12—C13—C8	−0.6 (4)
C7—C1—C6—C5	174.0 (2)	C9—C8—C13—C12	0.9 (4)
C8—N1—C7—C1	169.8 (2)	N1—C8—C13—C12	176.1 (2)

### Hydrogen-bond geometry (Å, °)

**Table:**

*Cg*1 is the centroid of the C1–C6 benzene ring.

**Table:**

*D*—H···*A*	*D*—H	H···*A*	*D*···*A*	*D*—H···*A*
O1—H1···N1	0.82	1.87	2.594 (3)	147
C9—H9···O1^i^	0.93	2.60	3.459 (4)	154.
C10—H10···*Cg*1^ii^	0.93	2.77	3.474 (3)	134
C13—H13···*Cg*1^iii^	0.03	2.80	3.501 (3)	133

Symmetry codes: (i) *x*, *y*, *z*−1; (ii) *x*, −*y*+3/2, *z*−1/2; (iii) *x*, −*y*+1/2, *z*+1/2.
